# The Influence of Different Types of Diabetes on Vascular Complications

**DOI:** 10.1155/2022/3448618

**Published:** 2022-02-22

**Authors:** Jiahua Wei, Jiaxing Tian, Cheng Tang, Xinyi Fang, Runyu Miao, Haoran Wu, Xiuge Wang, Xiaolin Tong

**Affiliations:** ^1^Changchun University of Chinese Medicine, Changchun 130117, China; ^2^Department of Endocrinology, Guang'anmen Hospital, China Academy of Chinese Medical Sciences, Beijing 100053, China; ^3^Beijing University of Traditional Chinese Medicine, Beijing 100029, China; ^4^Affiliated Hospital of Changchun University of Traditional Chinese Medicine, Changchun 130021, China

## Abstract

The final outcome of diabetes is chronic complications, of which vascular complications are the most serious, which is the main cause of death for diabetic patients and the direct cause of the increase in the cost of diabetes. Type 1 and type 2 diabetes are the main types of diabetes, and their pathogenesis is completely different. Type 1 diabetes is caused by genetics and immunity to destroy a large number of *β* cells, and insulin secretion is absolutely insufficient, which is more prone to microvascular complications. Type 2 diabetes is dominated by insulin resistance, leading to atherosclerosis, which is more likely to progress to macrovascular complications. This article explores the pathogenesis of two types of diabetes, analyzes the pathogenesis of different vascular complications, and tries to explain the different trends in the progression of different types of diabetes to vascular complications, in order to better prevent diabetes and its vascular complications.

## 1. Introduction

Diabetes mellitus (DM) is a chronic progressive disease characterized by systemic hyperglycemia. It is a principal health problem with increasing incidence worldwide. At present, there are approximately 463 million diabetic patients in the world, and on average, one in 11 adults (20-79 years old) has diabetes. In 2019 alone, 4.2 million adults died of diabetes and its complications [1].

Long-term complications of diabetes affect almost every organ of the body, especially vital organs such as the heart, brain, and kidney. Vascular complications caused by abnormal structures and functions of micro- and macrovessels are the most important cause of multiorgan dysfunction and increased morbidity and mortality from diabetic complications [2]. Diabetic nephropathy (DN) is one of the serious microvascular complications of diabetes. It is the main cause of renal failure in developed countries and developing countries, as well as the leading cause of death in diabetic patients. In 2017, there were more than 3.48 million diabetic nephropathy deaths worldwide, an increase of 145% compared to 1990 [3]. Diabetic retinopathy (DR) is the leading cause of vision loss in working-age adults (20-65 years) in developed countries [4]. And macrovascular complications mainly include coronary atherosclerotic heart disease and cerebrovascular events. Cardiovascular and cerebrovascular events are the main cause of disability and death in diabetic patients, accounting for more than 50% of the mortality rate of diabetic complications [5].

Type 1 and type 2 diabetes account for more than 90% of diabetes and are the most common types of diabetes. The pathogenesis of the two is not the same, that is, heterogeneity. Are there different development trends of vascular complications for different types of diabetes? In this review, we discuss the trend of vascular complications from the perspective of the pathogenesis of different types of diabetes.

## 2. Search Strategy and Selection Criteria

We systematically searched the Databases of Science, PubMed, EMBASE, MEDLINE, and Cochrane Library for articles published through to December 31, 2021. Search terms include the following: (“diabetes” or “diabetes” or “type 1 diabetes” or “T1DM” or “type 1 diabetes” or “type 2 diabetes” or “T2DM” or “type 2 diabetes”) and (“vascular complications” or “macrovascular complications” or “microvascular complications” or “diabetic retinopathy” or “diabetic nephropathy” or “coronary heart disease” or “diabetic cerebrovascular disease” or “diabetic foot”) and (“mechanism”). We have focused on the articles in the past five years. Of course, some articles with great significance have also been included, through the screening and classification of the searched articles, through careful reading of important articles, and using list display, summary, and analysis to draw conclusions.

## 3. Differences in Pathogenesis of Different Types of Diabetes Mellitus

### 3.1. Type 1 Diabetes

Type 1 diabetes mellitus (T1DM) is a chronic autoimmune disease with absolute deficiency of insulin secretion as the main manifestation [6], with a significant genetic predisposition and autoimmune destruction [7].

The basic cause of autoimmune diseases is the inability of the body to recognize autoantibodies from foreign antibodies, and this recognition depends on the major histocompatibility complex (MHC) on the cell surface. The major human histocompatibility complex, called human leukocyte antigen (HLA), is located on the short arm of human chromosome 6 and has the greatest impact on the development of diabetes, accounting for approximately 45% of disease susceptibility [8]. Two independent quantitative trait locus QTLs in the MHC gene regulate the ratio of CD4^+^ : CD8^+^ T lymphocytes in the general population, resulting in patients being more susceptible to immune diseases [9], which occurs mainly in HLA-DR3-DQ2, HLA-DR4-DQ8 haplotypes, or both [10]. This genetic manifestation is more obvious in younger adolescents with T1DM, that is, compared with adults, the prevalence of children is higher and the disease progresses more rapidly. This is because of the regulatory cytokine IL-10 secreted by CD4^+^ cells. It increases with age, the autoimmune response becomes more intense, and the destruction of *β* cells becomes more serious [11, 12]. Studies have shown that children who inherit DR3 and DR4 genotypes have a 55% risk of developing T1DM at the age of 12; the risk of autoantibodies is more than 60%, the incidence of autoantibodies positive in identical twins is more than 70%, and more than 60% probability of developing T1DM; and the concordance is inversely proportional to age; the younger the age, the higher the concordance [13, 14]. In addition, the prevalence of autoantibodies in overweight and obese adolescent patients was similar to that in nonobese healthy adolescents (2.18% and 1.86%), suggesting that obesity and insulin resistance do not increase the prevalence of insulin autoantibodies and the progression of islet autoimmune destruction [15].

**Figure 1:**
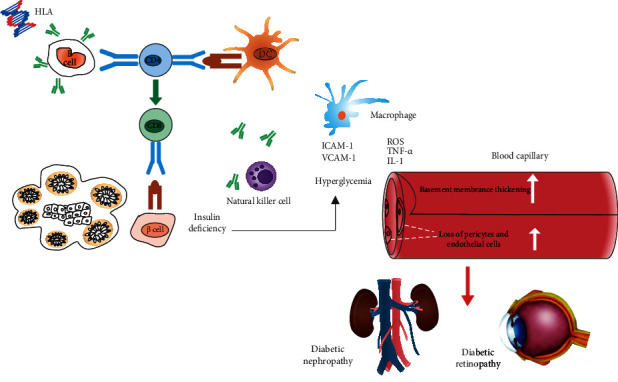
Mechanisms of T1DM and microvascular complications.

Immune mediation plays an important role in the development of T1DM especially the immune response centered on T lymphocytes [7], where B cells act as antigen-presenting cells presenting antigens to CD4^+^ and CD8^+^ T lymphocytes, producing antibodies that activate T lymphocytes and trigger or exacerbate islet autoimmunity [16, 17]. Diabetic CD4^+^ T cells isolated from nonobese diabetic type 1 (NOD) mice can clonally recognize epitopes formed by covalent cross-linking of insulinogenic peptides with other peptides in B cell secretory granules, called hybrid insulinotropic peptides (HIPs), which are key antigens for T cell immune responses and thus trigger islet autoimmune responses [18]. *β* cell progressive destruction is a key component of the absolute lack of insulin secretion and elevated blood glucose in T1DM. Under immune destructive stress, *β* cell subgroups will have senescence-related secretory phenotypes and senescence. Eliminating senescent cells can prevent immune-mediated *β* cell destruction in NOD mice, thereby delaying the progression of diabetes in mice [19]. Granulocyte-macrophage vinegar colony factor (GM-CSF) and IL-3 can play a role in maintaining immune homeostasis by promoting the effective phagocytosis of apoptotic cells by macrophages, and their functional defects will lead to an increase in macrophages, produce cellular inflammatory factors, and destroy autoimmunity [20]. In addition, pancreatic *β* cells overexpress IL-15 and Il-5*α* double transgenic mice, which appear similar to human insulin antibodies, which induce *β* cell destruction, while inhibiting IL-15 and IL-5*α* that can reverse the progression of diabetes [21] (see Figure 1).

The key mechanism of T1DM is genetic susceptibility and autoimmune response. Diabetic nephropathy, diabetic retinopathy, and T1DM have a common pathogenesis basis, namely genetic susceptibility and autoimmune response. Individuals with susceptibility genes produce autoantibodies against *β* cells and initiate an autoimmune response to *β* cells under the stimulation of external factors, leading to insulitis, progressive destruction of pancreatic *β* cells, and absolute insufficient insulin secretion, leading to increased blood sugar. In this process, immune cells including macrophages and natural killer cells play an important role. The massive activation of immune cells accelerates the mediated immune response. Hyperglycemia and activated immune cells release a large amount of proinflammatory factors and ROS, leading to increased vascular permeability, thickening of the vascular basement membrane, and damage to capillaries.

### 3.2. Type 2 Diabetes

Type 2 diabetes mellitus (T2DM) is characterized by insulin resistance and insulin secretion disorders [22, 23]. Many studies have demonstrated that insulin resistance precedes *β* cell defects [24, 25], which first occurs in skeletal muscle. Glucose transporter protein 4 (GLUT4) is an insulin-responsive glucose transporter protein [26, 27]. The mice that specifically knock out the GLUT4 glucose transporter have decreased insulin sensitivity in the muscles and liver and increased serum glucose and insulin in mice, showing severe insulin resistance and glucose intolerance. Impaired glucose transport stimulated by insulin is the reason for the decreased synthesis rate of muscle glycogen and liver glycogen [28–31].

Insulin resistance is also related to ectopic lipid deposition in the liver. The accumulation of hepatic diacylglycerol activates protease C-*ε* (PKC-*ε*), impairs insulin signal transduction, and inhibits the synthesis of liver glycogen and white adipocyte tissue, and the lipolysis of serotonin leads to disorders of lipid metabolism and increases cardiovascular risk [32–34]. The adipose cells play a regulatory role in the development of insulin resistance, which can produce adipokines [35], peptide hormones, including adiponectin (ACRP30), retinol binding protein-4 (RBP4), and resistin, as well as proinflammatory cytokines, such as interleukin- (IL-) 6 and tumor necrosis factor-*α* (TNF-*α*) [36]. Studies have found that high fasting plasma free fatty acids (FFA) in T2DM patients are usually elevated. A large amount of FFA stimulation leads to an increase in the production of highly reactive oxygen species (ROS) and reactive nitrogen species (RNS). High blood sugar and high FFA are common. It leads to the massive generation of ROS and oxidative stress and also activates stress-sensitive signal pathways, which in turn aggravates insulin resistance [37]. Foxo1 is a transcription factor that can increase the expression of key gluconeogenesis enzymes. Therefore, its upregulation leads to increased conversion of substrates into the liver to glucose. In the liver, insulin usually leads to the phosphorylation of Foxo1 function through the action of the protein kinase Akt and inhibition [38]. Insulin receptor substrates include insulin receptor substrate-1 (Irs1) and insulin receptor substrate-2 (Irs2), and Irs1 and Irs2 double knockout mice developed liver insulin resistance, which resulted in hyperinsulinemia and diabetes [39, 40].

**Figure 2:**
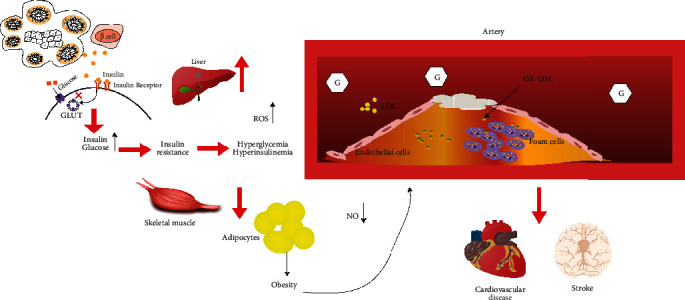
Mechanisms of T2DM and macrovascular complications.

Obesity is the main risk factor for insulin resistance [41]. Compared with normal-weight peers, fat insulin resistance is positively correlated with systemic and visceral obesity, fasting blood sugar, insulin resistance index HOMA-IR, and leptin [42]. In obese individuals, adipose tissue releases a large amount of nonesterified fatty acids and proinflammatory cytokines, thereby activating HIF-1*α*, causing adipose tissue dysfunction and inflammation; elevated fatty acid concentration can inhibit insulin-stimulated glucose transport activity, which can induce insulin resistance [33, 43, 44] (see Figure 2).

The key mechanism for the onset of T2DM is insulin resistance, and the main cause of diabetic macrovascular complications is atherosclerosis. Insulin resistance causes blood lipid metabolism disorder, and the absorption of glucose by fat cells increases, leading to obesity. Obesity and dyslipidemia are important risk factors for accelerating atherosclerosis. Hyperinsulinemia and hyperglycemia increase the release of ROS, leading to reduced nitric oxide (NO) activity, vasodilation disorders, damage to the vascular endothelium, and acceleration of vascular sclerosis. Plasma low-density lipoprotein (LDL) is oxidized to low oxidation under the action of ROS. Density lipoprotein (oxLDL) deposits on the arterial wall, forming arterial plaque.

## 4. Pathogenesis of Vascular Complications

### 4.1. Diabetic Nephropathy

Studies have shown that regardless of blood sugar control, diabetic patients have different risks of developing diabetic nephropathy, which may be due to their different genetic backgrounds, that is, the genetic predisposition to type 1 diabetic nephropathy [45, 46]. The immune inflammatory response caused by hyperglycemia may be the key to genetic susceptibility to diabetic nephropathy [47]. Long-term hyperglycemia puts the glomerulus in a state of hyperfiltration, leading to high perfusion and intraglomerular hypertension in the glomerulus, resulting in the proliferation and hypertrophy of the glomerulus, thickening of the basement membrane, and increased capillary permeability, which ultimately leads to glomerulosclerosis [48]. Hyperglycemia is an important cause of type 1 diabetic microangiopathy, the extracellular glucose concentration increases, and the capillary endothelial cells of the retina and the mesangial cells of the glomerulus cannot effectively regulate the entry of glucose into the cell, which leads to cell damage [49]. In addition, the advanced glycation end products produced by long-term hyperglycemia stimulate NF-*κ*B signal transduction to increase the production of antibodies and B cells [50]. Studies have confirmed that in a mouse model of T1DM, a large increase in glomerular and interstitial CD4^+^ and CD8^+^ T cells and deposition of immune complexes are found, local activated cytokine levels are increased to stimulate the activation of neighboring macrophages, and these complexes activate complement trigger inflammation and glomerulonephritis, leading to podocyte damage, thereby promoting the production of proteinuria [51, 52]. According to reports, increased release of oxygen free radicals from neutrophils in diabetic patients may damage endothelial cells and accelerate the progression of diabetic nephropathy [53].

The inflammatory factors tumor necrosis factor (TNF) and interleukin-1 (IL-1) may be involved in the development of diabetic nephropathy [54]. TNF-*α* enhances the expression and production of monocyte chemoattractant protein-1 (MCP-1) in human mesangial cells. Aggregated MCP-1 can recruit and activate giant cells in the glomeruli of proliferative glomerular disease. Phage cells cause glomerular structural damage by releasing proinflammatory and profibrotic cytokines [55–57]. In animal models of diabetic nephropathy, the renal expression of IL-1 is increased [58]. This feature is related to the increased expression of proinflammatory molecules such as intercellular adhesion molecule 1 (ICAM1), vascular cell adhesion protein 1 (VCAM1), and chemokines [11, 12]. And IL-1 is related to the dysfunction of hyaluronic acid produced by the proximal tubular epithelial cells of the kidney and directly increases the permeability of vascular endothelial cells [59]. NETosis is a unique form of cell death. It is an important core component of the innate immune system. Activated neutrophils capture and kill pathogens by releasing extracellular cellular capture nets (NETs) composed of depolymerized chromatin and intracellular granule proteins [60]. NETosis plays an important role in the development of diabetes and its vascular complications. Under hyperglycemia, neutrophils are more prone to cause NETosis, thereby damaging blood vessels and inhibiting wound healing [61, 62].(see Figure 1).

### 4.2. Diabetic Retinopathy

A Finnish study showed that the pathogenesis of proliferative diabetic retinopathy (PDR) has a significant genetic tendency, with a heritability rate of more than 50% [63]. The frequencies of allele C and homozygous CC in patients with diabetic retinopathy are significantly higher than those in nonretinopathy patients, and the risk of retinopathy in homozygous genotype CC is increased [64]. Another study also found that CACNB2 is a susceptibility gene for diabetic retinal proliferative diseases and CACNB2 is abundantly expressed in retinal cells, encoding the *β*2 subunit of the L-type calcium channel and regulating the expression of VEGF [65]. Proliferative diabetic retinopathy only occurs in patients with T1DM who are genetically deficient in glucose-6-phosphate dehydrogenase (G6PD). The genetic defect of G6PD leads to impaired activity of the pentose phosphate pathway of glucose metabolism, thereby accelerating diabetic microvascular complications [66]. HLA plays an important role in immune response and immune tolerance and is an important mechanism for the pathogenesis of T1DM. Similarly, it plays an important role in retinopathy. The frequency of HLA-B62, HLA-Cw4, and HLA-DQ4 in the PDR group was significantly higher than that in the non-DR group. HLA-B62 and HLA-Cw4 form haplotypes with HLA-DR and HLA-DQ antigens, thereby promoting PDR occurrence and progress [67].

With the prolonged course of the disease, the rate of *β* cell destruction is an independent risk factor for diabetic retinopathy. The duration of chronic hyperglycemia and diabetes is the most important factor associated with retinopathy [68]. The mutation rate of glycated hemoglobin is associated with early retinopathy and increased proteinuria excretion [69]. The DCCT/EDIC studies have shown that elevated average glycosylated hemoglobin is the main risk factor for diabetic retinopathy, and intensive hypoglycemic therapy can significantly reduce the progression of type 1 diabetic retinopathy [70]. The development of T1DM into microvascular disease may also be related to gender, and the delayed onset of menarche increases the risk of diabetic nephropathy and diabetic retinopathy [71]. Genetic variation may interact with epigenetic variation, thereby increasing the risk of disease. Studies have found that the differential DNA methylation of close to 233 unique genes is related to PDR. The mechanism of PDR may be the resulting interaction between genetic variation and epigenetic variation [72]. Compared with patients without microvascular complications, the expression of serum miR-518d-3p and miR-618 in patients with diabetic retinopathy is upregulated [73]. Two miRNAs related to angiogenesis, miR-27b and miR-320a, are related to the incidence and progression of retinopathy [74, 75]. miR-320a regulates glycolysis and inhibits angiogenic factors, and miR-27b is believed to promote angiogenesis by targeting anti-angiogenic genes [76] (see Figure 1).

### 4.3. Coronary Heart Disease

Cardiovascular disease is the most common cause of death in diabetes, accounting for more than 50% of the death rate of T2DM [77]. Insulin resistance increases the incidence of atherosclerosis in patients with T2DM, which is the main reason for the development of macrovascular disease; the main feature of diabetic macrovascular disease is the imbalance of vascular homeostasis caused by dysfunction of endothelial cells and smooth muscle cells, which ultimately leads to atherosclerotic thrombosis [78, 79].

Vascular endothelial dysfunction is one of the manifestations of changes in the arterial wall caused by metabolic abnormalities caused by insulin resistance; it is closely related to cardiovascular events and is an important early event in the pathogenesis of atherosclerosis [80–82]. Endothelial cell product nitric oxide (NO) is an important regulator of vascular tone, which can inhibit the proliferation of vascular smooth muscle cells, effectively inhibiting platelet aggregation and the expression of adhesion factors VCAM-1 and ICAM-1 in the blood vessel wall [83, 84]. Long-term hyperglycemia will cause the continuous production of intracellular ROS. Driven by hyperglycemia and oxidative stress, increased ROS levels trigger insulin resistance and accelerate the production of advanced glycation end products (AGEs) [85]. Under the combined action of ROS and AGEs, NO is inactivated, leading to vasodilation disorders, increasing vascular permeability, promoting the increase of endothelial factor expression of endothelial cell adhesion molecules, and accelerating vascular sclerosis; under oxidative stress, the plasma low-density lipoprotein (LDL) is oxidized into oxidized low-density lipoprotein (oxLDL) and deposited on the arterial wall [86–89]. The inactivation of NO also leads to the expression of MCP-1, which recruits monocytes, and the monocytes transform into lipid foam cells, which deposit on the arterial wall and form arterial plaques [90]. Long-term hyperglycemia can also increase the production of mitochondrial superoxide, thereby activating protein kinase C and inhibiting the activity of nitric oxide synthase (eNOS), thereby accelerating atherosclerosis [91]. The excessive activation of the nicotinamide adenine dinucleotide phosphate (NADPH) oxidase of the Nox family produces excessive ROS, which promotes the development of arteriosclerosis, while the Nox1 gene of knockout female mice delays this progress [92]. In addition, interleukin-1*β* (IL-1*β*) has long been regarded as an inflammatory cytokine, which is closely related to the pathogenesis of T2DM and atherosclerosis. Inhibiting IL-1*β* may delay the progression of cardiovascular disease [93, 94].

Obesity is the main risk factor for diabetes and coronary heart disease, and it is also the link between the two. Relevant data suggest that severe obesity (BMI > 35 kg/m^2^) can increase the risk of diabetes and coronary heart disease by approximately two times [95]. Compared with patients who have not undergone bariatric surgery, obese diabetic patients undergoing bariatric surgery have a 65% reduction in the risk of death from cardiovascular disease and a reduction in all-cause mortality [96]. Insulin resistance can lead to disorders of lipid metabolism and can lead to obesity. On the contrary, obesity further aggravates insulin resistance. Obesity leads to ectopic fat deposition and lack of insulin signal transduction, triggers insulin resistance, inhibits liver glycogen synthesis, and increases liver gluconeogenesis, especially in peripheral tissues such as fat cells, leading to abnormal lipid metabolism [97, 98]. Insulin is the main regulator of fat content in adipocytes. It is both an effective inhibitor of lipase and an important activator of lipoprotein lipase. It can enhance the uptake of FFA and the synthesis of triglycerides in adipocytes [99]. However, insulin resistance reduces the activity of lipoprotein lipase, increases FFA, and continues to produce an atherogenic phenotype [100] (see Figure 2).

### 4.4. Diabetic Cerebrovascular Disease

A meta-analysis of 102 prospective studies showed that diabetes increased the risk of stroke and provided a twofold increased risk of cerebrovascular disease [101, 102]. Stroke is a major macrovascular complication of diabetic cerebrovascular, and hyperglycemia makes the risk of stroke greater, especially ischemic stroke [103]. In the case of hyperglycemia, acidosis inside and outside the brain cells causes damage to neurons and glial cells, resulting in swelling of endothelial cells and narrowing of the blood vessel lumen and cerebral ischemia [104, 105]. In addition, hyperglycemia can also induce superoxide produced by the activation of NADPH oxidase in brain neurons, triggering neuronal death [106, 107].

The stenosis of the vascular lumen caused by atherosclerosis of the large intracranial arteries is one of the most common causes of stroke worldwide [108]. Studies have shown that the formation of atherosclerotic plaque is related to the blood hypercoagulable state. Cerebral artery plaque and blood hypercoagulable state together increase the risk of ischemic stroke recurrence and death [109, 110]. Insulin resistance is closely related to stroke. An ARIC study on the risk of atherosclerosis in the community found that for every 50 pmol/L increase in fasting insulin of nondiabetic patients, the relative risk (RR) of ischemic stroke will increase [111]. Furthermore, another cohort study on the risk of stroke in elderly patients with diabetes and patients with no diabetes also showed that elevated fasting insulin levels are a risk factor for stroke [112]. The metabolic disorder caused by insulin resistance accelerates the process of atherosclerosis, and it plays an important role in the pathogenesis of ischemic stroke [113, 114]. A study confirmed that the protein C (PC) and protein S (PS) levels of patients with ischemic stroke insulin resistance were significantly lower than those of noninsulin resistant patients [115]. And the liver coagulation factors F8 and F9 and human fibrinogen gamma chain FGG of insulin resistant subjects are highly expressed, which enhances platelet activation, adhesion, and aggregation, leading to a hypercoagulable state of blood [116]. In addition, insulin resistance can also induce hepatocytes and endothelial cell lines to increase the expression and secretion of plasminogen activator inhibitor 1 and aggravate thrombosis [117].

Insulin resistance and hyperinsulinemia have been shown to be closely related to the increased incidence of cardiovascular disease and hypertension [118]. And hypertension increases the incidence of hemorrhagic and nonhemorrhagic strokes [119]. Studies have shown that early stroke progression is related to systolic blood pressure [120], and compared with nonhypertensive and nondiabetic patients, diabetic patients with blood pressure≧160/95 mmHg have a 4-fold increase in the risk of stroke incidence and an 8-fold increase in the risk of stroke-related mortality [121]. Hypertension and insulin resistance make patients with T2DM more susceptible to ischemic stroke [122]. In the setting of insulin resistance, insulin's stimulation of NO biological activity is reduced, that is, endothelial cell NO synthase activation is reduced, NO destruction is increased, vasoconstriction is caused, blood pressure is increased, and stroke progression is accelerated [123] (see Figure 2).

### 4.5. Diabetic Foot

The data show that more than one-fifth of diabetic patients will eventually develop diabetic foot, and at least one-quarter of the wounds and ulcers cannot heal, putting patients at risk of amputation [124]. A study on the prevalence of diabetic foot in Japanese showed that among Japanese type 2 diabetic patients, the incidence of diabetic foot and amputation due to diabetic foot were 0.3% and 0.05%, respectively, and compared with patients without a history of diabetic foot, the mortality rate of diabetic foot patients has increased significantly by about two times [125]. And what is even more shocking is that the duration of diabetic foot is positively correlated with mortality; the ten-year mortality rate of the disease has increased by about three times compared with the five-year period [126].

Diabetic foot is the result of a variety of risk factors, including paraesthesia caused by peripheral neuropathy and ischemia caused by peripheral arterial disease, and about 50% of diabetic foot patients have peripheral arterial disease [127]. The factors that lead to neuropathy and lower extremity vascular disease will accelerate the progress of diabetic foot to a certain extent. In a hyperglycemic environment, prolonged inflammation in the microcirculation can lead to the thickening of the capillary basement membrane, transparent arterioles, impairing of the normal movement of nutrients, and activated white blood cells between the capillary cavity and the interstitium, leading to vasodilation ability during local injury restriction, causing ischemia. Long-term hyperglycemia and insulin resistance inhibit the production of NO by blocking the activation of NOS in endothelial cells and produce more ROS, which accelerates the process of vascular endothelial damage and arteriosclerosis [128]. And the existence of insulin resistance can cause inflammation infiltration on the surface of the lesions in high-fat-fed mice, while at the same time impairing collagen deposition and prolonging wound healing time [129].

Macrophages, one of the immune cells, are the central cells of the inflammatory response and are considered the main regulator of wound healing [130]. Studies have found that the phenotypic transition of macrophages from M1 to M2 in diabetic foot ulcer wound tissue is defective, and the recruitment of immune neutrophils and macrophages is reduced, which delays the time of wound healing and aggravates the risk infection of diabetic foot [131–134]. In addition, another study analyzed the distribution and phenotype of peripheral blood T cells in healthy controls, diabetic patients, and diabetic patients with foot ulcers. The results showed that diabetic patients, especially those with foot ulcers, had a significant decrease in the number of naive T cells and a decrease in the diversity of T cells antigen receptors (TCR) [135]. These data indicate that the occurrence of diabetic foot is also related to immune mechanism. In addition, the incidence of diabetic foot is also closely related to microvascular complications [125]. A study in the Nordic region showed that almost most patients with diabetic foot ulcers suffer from diabetic retinopathy, of which proliferative diabetic retinopathy is the majority, accounting for about one-third of the total population [136], and the presence of diabetic retinopathy and proteinuria significantly increases the risk of the development of diabetic foot ulcers [137].

## 5. Discussion and Conclusion

Diabetes mellitus is a chronic and progressive metabolic disease, and the complications are the main cause of death of diabetes and the result of the chronic progression of diabetes. Damage to pancreatic islet function leads to absolute and relative lack of insulin secretion, which keeps the body in a state of hyperglycemia for a long time. On one hand, the long-term stimulation of high blood sugar in various tissues and organs increases the body's antibodies, accelerates the deposition of immune complexes, and triggers the immune inflammatory mechanism. On the other hand, hyperglycemia increases the accumulation of inflammatory factors and continues to activate the oxidative stress response. The above-mentioned pathological conditions have exacerbated the tendency of diabetes to progress to complications. Research data shows that the prevalence of complications in diabetic patients increases significantly with the increase of the duration of the disease [138], and once diabetes develops into a complication, drug treatment is difficult to reverse. It can be seen that the complications of diabetes are the focus of clinical treatment and are also the difficulties that need to be overcome urgently.

Long-term hyperglycemia affects various tissues and organs of the body, and diabetic nephropathy, diabetic retinopathy, coronary heart disease, stroke, and other vascular complications not only directly lead to the increase in disability and mortality of diabetic patients but also bring a heavy economic burden to patients. Studies have shown that patients with T2DM have an increased risk of cardiovascular disease, and the disease prognosis is worse when cardiovascular complications occur [139]. Once diabetes progresses to vascular complications, the disease is complicated, the treatment becomes more difficult, and the burden of the disease increases, which seriously affects the prognosis of patients.

Vascular complications mainly include microvascular and macrovascular complications. Damage to large blood vessels and capillaries involves multiple organs such as the heart, brain, kidneys, eyes, and feet. Microvascular complications mainly include diabetic nephropathy and diabetic retinopathy. Studies have shown that diabetic nephropathy and diabetic retinopathy have significant genetic predispositions, among which diabetic proliferative retinopathy has the highest genetic probability. Immune destruction is a key factor that directly affects the progress of the two. A large number of immune complexes are deposited, activate local immune cells, and produce an immune inflammatory response. At the same time, as a large number of *β* cells are destroyed, the infiltration of high blood sugar leads to the release of large amounts of inflammatory factors. Under the immune-mediated interaction, capillary permeability increases, and the basement membrane thickens. In addition, it is interesting that the existence of proliferative diabetic retinopathy is closely related to diabetic nephropathy. Does this association also indirectly indicate that the two have a common basis? Coronary heart disease, stroke, and diabetic foot are the main macrovascular diseases. The basic pathological change is atherosclerosis. The main cause of T2DM is insulin resistance, which in turn is the main cause of atherosclerosis. First, insulin resistance causes glucose and lipid metabolism disorder, and the absorption of glucose by fat cells increases, leading to obesity. Obesity and dyslipidemia are also important risk factors for atherosclerosis. Secondly, hyperinsulinemia and hyperglycemia under the compensation of insulin resistance can damage the endothelium of large blood vessels, stimulate smooth muscle proliferation, and accelerate the process of atherosclerosis. From the above analysis, we can infer that T1DM is more likely to progress to microvascular complications, while T2DM is more likely to progress to macrovascular complications.

Early intervention and treatment of diabetes and its complications are an urgent need to cope with the increasing number of diabetic patients today, and they are the focus of clinical urgent need to overcome. The heterogeneity of the pathogenesis of T1DM and T2DM affects the tendency of the two to develop into different vascular complications. It reminds us that in the process of clinical diagnosis and treatment of different types of diabetes, we should implement targeted treatment plans. Early intervention for related vascular complications can delay the progression of diabetic vascular complications. However, current studies, including large-scale clinical studies and basic experiments, mostly focus on the study of their respective pathological mechanisms and complications, and fail to integrate the pathological mechanisms and complications of different types of diabetes to explore organically and reveal the differences in their evolutionary process trend. This may become a new direction of attention and research in the future.
